# Application of chia (*Salvia hispanica*) seeds as a functional component in the fortification of pineapple jam

**DOI:** 10.1002/fsn3.819

**Published:** 2018-10-16

**Authors:** John M. Nduko, Rita W. Maina, Rose K. Muchina, Simon K. Kibitok

**Affiliations:** ^1^ Department of Dairy and Food Science and Technology Egerton University Egerton Kenya

**Keywords:** chia seed, food fortification, functional foods, pineapple jam

## Abstract

This study aimed at producing chia seed‐fortified pineapple jam and evaluation of its physicochemical and sensory characteristics. Five pineapple jam formulations were developed, starting with the basic formulation (jam 1/control) containing sucrose and added pectin. The other jams (jam 2, jam 3, jam 4, and jam 5) had sucrose and pectin but with chia seeds added at rates of 6.25, 12.5, 25, and 50% w/w, respectively. Crude protein of the jams was analyzed through macro Kjeldahl method, and the crude fiber was estimated by the Weende method. Thirty‐two semi‐trained panelists performed sensory evaluation test of the developed jams using 5‐point hedonic scale. Chia pineapple jam had golden color compared to control pineapple jam, which had yellow color. The fresh chia pineapple jams had significantly (*p* < 0.05) different protein and crude fiber contents in each sample. The protein content in the control was 0.53%, while it ranged between 1.60 and 8.60% for the chia seed‐fortified jams. For crude fiber, the values were 4.83% for the control and 5.38, 9.08, 13.33, and 21.02% for jam 2, jam 3, jam 4, and jam 5, respectively. General acceptability and sensory evaluation (flavor, color, and texture) showed significant (*p* < 0.05) differences compared to the control, while spreadability had no significant (*p* > 0.05) differences. The information obtained from this study indicates that chia‐fortified pineapple jam could be produced with favorable sensory attributes that could be used for jam making and other processed products to benefit from the functional components in the chia seeds.

## 1 INTRODUCTION

Jam is a type of a fruit preserve/spread usually made from all types of fruits, sugar, and pectin for consumption during the off‐season. Jams are famous mainly due to their availability, organoleptic properties, and low cost that are usually canned or sealed after production in sterilized bottles (Muresan, Pop, Muste, Scrob, & Rat, 2014). Jam was traditionally prepared to preserve seasonal fruits by heating them with sugar and pectin in the fruit (Patrick, 1982). Currently, fruit preserves are made both commercially and in home‐based operations. The jam is prepared by chopping the fruits then followed by cooking with sugar and pectin until the required consistency is achieved, after which it is packed in sterilized jars or cans (Ihekoronye, 1999).

Pineapple is one fruit that is used for making jam that is an excellent source of vitamin C and manganese among other nutrients. In 100 g, the fruit contains 12 g carbohydrates, 0.2 g fat, 1.2 g protein, and 1.4 g fiber (Moreiras, Carbajal, Cabrera, & Cuadrado, 2001). However, when pineapple is processed into jam, the percentage of nutritional content drops significantly resulting into trace fat and concentration of carbohydrates, which mainly comprises of sugar. Moreover, jams normally have lower vitamin C content compared to their fresh fruits counterparts as a result of the heat to which they are exposed during processing (Naeem et al., 2017). One approach to the solving of the problem of nutrient loss is to fortify the jam with nutrient‐dense food products. Through fortification, a processor can replenish lost nutrients during processing or enrich the nutritional value of a food by adding extra nutrients. For example, Toves (2004) demonstrated the fortification of jam with soluble dietary fiber and Lavelli, Vantaggi, Corey, and Kerr (2010) fortified apple puree with green tea extract equivalent to that present in a cup of green tea. Jams can therefore be fortified as well as used as delivery vehicles for a number of nutrients including functional components. Functional components in food are chemicals that have physiological benefits and/or reduce the risk of chronic disease beyond basic nutritional functions (Ansari & Kumar, 2012). Among the foods that have functional components are chia (*Salvia hispanica*) seeds. These are unprocessed, ready to eat, whole grain foods containing up to 39% oil which has the highest known content of α‐linolenic acid (ω‐3) of up to 68% compared to other natural sources (Porras‐Loaiza, Jiménez‐Munguía, Sosa‐Morales, Palou, & López‐Malo, 2014). These healthy omega‐3 fatty acids aid in reduced risk of cardiovascular diseases. Chia seeds also contain carbohydrates (26%–41%), dietary fiber(18%–30%) that aids in reducing the risk of type 2 diabetes, proteins (15%–25%), antioxidants such as gallic acid, which fight free radicals in the body that cause degenerative diseases, a high amount of vitamins, and minerals such as calcium (Timilsena, Adhikari, Barrow, & Adhikari, 2016). Chia seeds are also free of cholesterol and trans fats, and limited allergen tests have shown no adverse reactions to chia seeds (Ayerza & Coates, 2004).

Chia seeds can also be added to processed foods as an additional ingredient in a number of dishes. Chia seed is fast gaining popularity worldwide due to increasing desire to change to healthier lifestyles and increased incidence of cardiovascular diseases, type‐2 diabetes, colorectal cancer, obesity, and other related illnesses (Coelho & Salas‐Mellado, 2015). On the other hand, chia seeds are being used as healthy oil supplements, for example, in cake formulations (Borneo, Aguirre, & Leon, 2010), it is commonly consumed as a salad from chia sprout in beverages, cereals, and salads. The European Commission (European Novel Foods Regulations) approved the use of chia seeds in bread products at a rate of not more than 5% (EC, 2009). Other applications include its use in breakfast cereals, cookies, fruit juices, yogurt (Attalla & El‐Hussieny, 2017), and jams.

A normal fruit jam has an even consistency without distinct fruit pieces, bright color, good fruit flavor, and semi‐jelled texture; is easy to spread; and has no free‐flowing liquid (Berolzheimer, 1969). Fortification of jam can therefore affect the taste/mouthfeel or impart grittiness and bitterness to the product and thus overall acceptability. Fortification is sensitive in particular where the texture of a product such as jam needs to be preserved. The aim of this study was to fortify pineapple jam with chia seeds and evaluate its sensory characteristics and acceptability. We therefore prepared pineapple jam mixed with different concentrations of chia seeds. Proximate analysis and sensory data of the prepared jams indicate that pineapple jam could be fortified and demonstrate a potential application of chia seeds for maximum benefit from its functional properties.

## 2 MATERIALS AND METHODS

### 2.1 Materials and reagents

Fresh pineapple fruits having uniform maturity were purchased from Nakuru market, Kenya. Chia seeds, sugar, and citric acid were bought from Nakumatt Supermarket Ltd (Nakuru, Kenya). Equipment, refractometer, pH meter/scale, knives, metal buckets, mixer, cooker, and sterile containers were obtained from the Department of Dairy Food Science and Technology (DAFTECH) food pilot plant of Egerton University, Kenya. Laboratory reagents for protein and crude fiber analysis were obtained from Kobian Ltd (Nairobi, Kenya).

### 2.2 Chia‐fortified pineapple jam production

After assembling the necessary equipment, the pineapples were washed thoroughly using running tap water to remove any dirt that was on the surface, the pineapples were then trimmed, peeled, and decored. The peels were placed separately for further washing as they were to be used as pectin. The pineapples were sliced then pulverized using a household blender to obtain fruit pulp, which was collected in a pre‐weighed glass jar. The °Brix of the fruit pulp was measured using a refractometer and pH by pH meter. The pineapple peels were washed thoroughly, pulverized using a household blender to extract pectin, and harvested in a clean metal container then added at a rate of 1%. The ingredients were placed in an oven to cook (the fruit pulp and pectin together) stirring while adding sugar (up to 50%) for 3 hr until the jam obtained the desired consistency. The jam was emptied into five different sterile containers; each was filled with 500 ml of the jam. Chia seeds at different concentrations (6.25, 12.5, 25, and 50% w/w) were added to three (triplicates) of the jam‐filled containers, and three containers were left with no chia seeds as control samples.

### 2.3 Chemical analysis

#### 2.3.1 Determination of crude protein

Proteins analysis of chia‐fortified pineapple jam was conducted using Kjeldahl method (method number 920.152) according to the Association of Official Analytical Chemists (AOAC) protocol (AOAC, 2000). Briefly, 1 g of each sample was measured using a weighing scale and was each carefully inserted into clean Kjeldahl flasks, and labeled. About 2 g of catalyst was then added into each flask, and then 10 ml of concentrated sulfuric acid was added into each flask. The sample was placed into the digester unit and allowed to digest for 3 hr at a temperature of 360°C until the color changed from black to light green. After digestion, the samples were allowed to cool before removing them from the protein digester unit and then were cooled under running tap water. The samples were then transferred into conical flasks, and 50 ml of distilled water was added into each sample. After digestion, the samples were then distilled using a distiller. Fifty milliliters of boric acid was prepared and put in a 250‐ml volumetric flask after which 10 ml was pipetted into a conical flask, and 4–5 drops of mixed indicator was added. This procedure was repeated for all the five treatments. Ten milliliters of 40% sodium hydroxide solution was then added into each flask, and 10 ml of the digested sample containing distilled water was added into the flask containing sodium hydroxide. This was repeated for all the samples. The Kjeldahl flask containing 10 ml sodium hydroxide and 10‐ml sample was adjusted into the distillation unit. A receiving tube was put under the conical flask containing 4% boric acid and mixed indicator, and the process allowed to distill for 5 hr until the color of indicator changed from brown to green and about 150 ml of distillate was obtained. The procedure was repeated for all the samples. After distillation, the ammonia was absorbed by boric acid, and then the distillate titrated using standard 0.1 N HCl until the color changed from green to pink. The results of titration were recorded, and the titration procedure was repeated for all samples. The results obtained were used to calculate the percentage protein in each sample using formulae (1):

(1) $$\%\;\text{Nitrogen}=\frac{0.014\times \text{Normality of HCL}\times \text{Volume of HCL required}\times 100}{\text{Weight of sample}}$$

Thus, % protein = % nitrogen × 6.25 (conversion factor for fruit products).

#### 2.3.2 Determination of dietary fiber

Crude fiber analysis was conducted using the Weende method (Pearson, 1996). Ten grams of each sample was weighed and put in glass beakers. A 100 ml of water was added, and then 2.04 N of dilute sulfuric acid was added. The volume of hot water was increased to 200 ml and let to boil for 30 min. The residue was then removed, filtered, and washed thrice with hot water. The washed residue was digested with 25 ml of 1.78 N potassium hydroxide and let to boil for 30 min, and then it was filled and washed thrice. It was then dried in an oven at 105°C, cooled then weighed until it attained a constant weight, and recorded. The residue was placed in a furnace (muffle) to ash at 550°C then cooled and weighed. The fiber was determined as in the formulae (2).

(2) $$\%\;\text{Fiber}=\frac{\text{Weight of sample after oven drying}-\text{Weight of ash}}{\text{Weight of sample}}\times 1000$$

#### 2.3.3 pH and Brix determination

The pH of the pineapple jam samples was obtained as described by Egan, Kirk, and Sawyer (1985). The total soluble solids in the final jam were measured using a refractometer. The refractometer was first set to 0° Brix using 1 ml distilled water, since the refractometer in the laboratory had a maximum limit of 30°, we diluted 5 ml of each sample with 10 ml of distilled water to acquire a ratio of 1:2 then measured the Brix then the readings recorded.

### 2.4 Sensory evaluation

Sensory evaluation was conducted in isolated booths at the Dairy and Food Science and Technology laboratory. Thirty‐two semi‐trained personnel conducted the analysis. Each panelist was given plastic cups filled with the samples that were coded using three alphabets codes, and each sample was tested in triplicate. Before the analysis, the entire panelists were given oral instruction on the attributes (color, flavor, texture, spreadability, and general acceptability) and the direction of testing but randomly. They were also provided with a palate cleanser (water) and a slice of bread for spreadability, and also, scorecards were provided to each panelist. The score sheet contained a five‐point hedonic scale, with the key words rating: dislike extremely (1), dislike (2), neither like nor dislike (3), like (4), and like extremely (5).

### 2.5 Statistical analysis

Analysis of variance: Protein and fiber content, sensory characteristics (color, texture, and flavor), spreadability, and general acceptability data provided by the panelists were subject to one‐way ANOVA, using chia seed content (%) as fixed factor. The mean ratings and honestly significant differences were determined based on Tukey's test (*p* < 0.05).

## 3 RESULTS

### 3.1 Jam preparation

**Figure 1:**
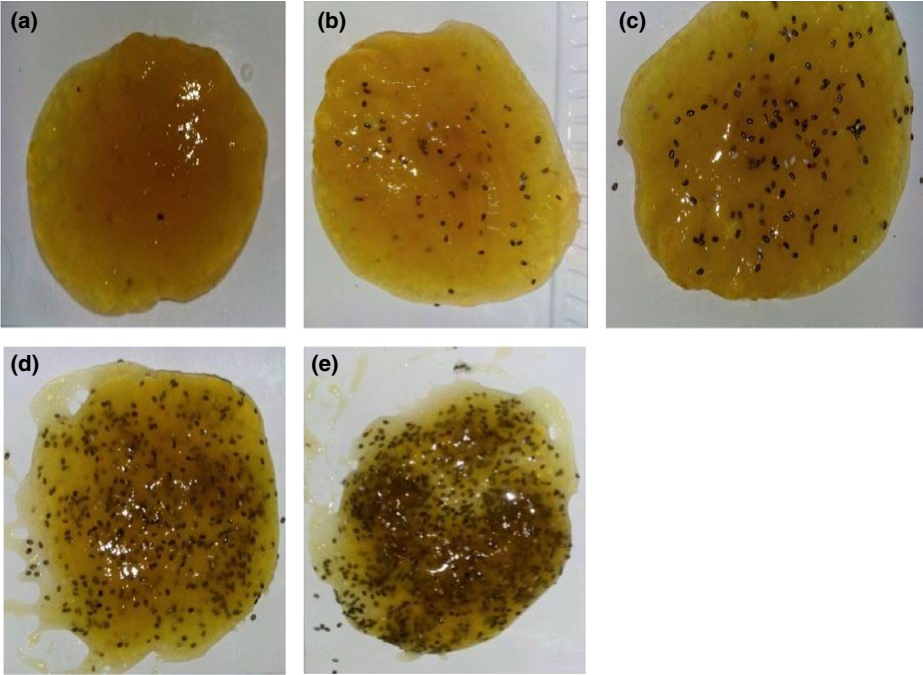
Photos of chia seed‐fortified jams. (a) Control; (b) 6.25%; (c) 12.5%; (d), 25%; and (e), 50% chia seeds

Figure 1 shows images of pineapple jam products made from pineapple fruit pulp, sugar, pectin, and chia seeds. Chia‐fortified pineapple jam had golden color compared to control pineapple jam, which had yellow color (Figure 1a).

### 3.2 Proximate analysis

The total protein content of pineapple jam (control) was 0.53% and that of the four chia seed‐fortified jams varied between 1.60% and 8.60% depending on the amount of chia seeds added (Table 1). To find out differences in protein content among the jam formulations, one‐way ANOVA was conducted. The *p*‐value corresponding to the *F*‐statistic of one‐way ANOVA was lower than 0.05, which suggested that the protein content in one or more jam formulations was significantly different. Tukey's HSD test was applied to the jam formulations. It was found out that there were significant differences (Tukey's HSD *p*‐value < 0.001) in protein content between all the jam formulations. The fiber content of the jams formulated also increased with the amount of chia seeds added (Table 1). The means of fiber content were also subjected to one‐way ANOVA. The *p*‐value corresponding to the *F*‐statistic was lower than 0.01, which indicated significant differences in fiber content among the different jam formulations. Similar to the protein content, it was found that there were significant differences (Tukey's HSD *p*‐value <0.001) in fiber content between all the jam formulations (Table 1).

**Table 1:** Proximate analysis of chia seed‐fortified pineapple jams

Trial	Added chia seeds (%)	Protein (%)	Fiber (%)	pH	Brix (°)
A	0	0.53 ± 0.03^a^	4.83 ± 0.08^f^	3.5	68
B	6.25	1.60 ± 0.03^b^	5.38 ± 0.06^g^	3.2	69
C	12.50	2.35 ± 0.09^c^	9.08 ± 0.09^h^	3.2	68
D	25.00	4.65 ± 0.09^d^	13.33 ± 0.08^i^	3.4	68
E	50.00	8.60 ± 0.11^e^	21.02 ± 0.09^j^	3.7	69

The protein and fiber values are means ± *SD* from three replicates. Different superscript alphabets in one column indicate significant differences among mean values according to the Tukey's test (*p* < 0.05).

### 3.3 pH and Brix

The sample with 0% chia seeds (control) had a pH of 3.5, while the samples fortified with chia seeds had pH ranging between 3.2 and 3.7 (Table 1). For the Brix, the control sample had a Brix of 68°, while sample fortified with chia seeds had a Brix content of 68–69°^ ^(Table 1).

### 3.4 Sensory evaluation

The addition of chia seeds led to significant changes (*p* < 0.05) in the sensory attributes that are evaluated by the tasting panel as shown in Table 2. Results showed that the addition of 6.25% chia seeds did not vary the jam color and texture significantly, while the addition of higher levels of chia seeds resulted into significant changes (*p* < 0.05). On the other hand, introduction of chia seeds into the jam significantly changed the flavor and the general acceptability of the jam while there were insignificant (*p* > 0.05) changes in jam spreadability by incorporating chia seeds into the jam. Although the addition of chia seeds at 6.25% and 12.5% levels decreased the ratings of the jams, the scores indicated moderate acceptance of the products. However, addition of chia seeds at 25% and 50% levels adversely affected the sensory quality of jam, a fact that negatively impacted consumer's general acceptability.

**Table 2:** Mean sensory evaluation of chia seed‐fortified pineapple jams and control (pineapple jam)

Attribute/Trial	A	B	C	D	E
Color	3.69 ± 0.07^a^	3.91 ± 0.13^a^	3.00 ± 0.31^b^	2.69 ± 0.09^bc^	2.34 ± 0.06^c^
Texture	4.00 ± 0.13^a^	4.09 ± 0.06^a^	2.59 ± 0.08^bc^	2.87 ± 0.05^c^	2.31 ± 0.10^b^
Flavor	4.38 ± 0.11^a^	3.88 ± 0.05^b^	3.81 ± 0.07^b^	3.56 ± 0.17^b^	3.75 ± 0.04^b^
Spreadability	3.96 ± 0.12^a^	4.00 ± 0.09^a^	4.03 ± 0.06^a^	3.9 ± 0.03^a^	4.06 ± 0.05^a^
General acceptability	4.06 ± 0.08^a^	3.53 ± 0.03^b^	3.59 ± 0.05^b^	2.59 ± 0.05^c^	2.65 ± 0.04^c^

The sensory attribute values are means±standard deviations from three replicates. Different alphabets in one row indicate significant differences among mean values according to the Tukey's test (*p* < 0.05). Trials A, B, C, D, and E represent the control, 6.25%, 12.5%, 25%, and 50% w/w additions of chia seeds, respectively.

## 4 DISCUSSION

Many fruits are used for the preparation of jams, which are popular because of their low cost, all year long availability, and organoleptic properties (Gałkowska, Fortuna, & Prochwicz‐zagórska, 2010). The addition of chia seeds did not affect the gelling of jam as was evident with jam spreadability scores of the formulated jams. The control jam had a protein content of 0.53% that is similar to the 0.46% protein content that was reported by Eke‐Ejiofor and Owuno (2013). The protein content in pineapple jam remains low since none of the used ingredients (fruits, sugar, pectin, and citric acid) are good sources of proteins and fat. On the other hand, the addition of chia seeds increased the protein content proportional to the amount added. Chia seeds contain approximately 16.5% proteins, which is higher compared to the most commonly consumed grains such as wheat, barley, and oats (Timilsena et al., 2016; Valdivia‐López & Tecante, 2015). Studies have also shown that chia seeds contain both essential amino acids and non‐essential amino acids such as methionine, phenylalanine, cysteine, and glycine in significant amounts, giving it a high protein quality score (Ding et al., 2018; Nitrayová et al., 2014), and hence, it can be used to supplement low protein quality products such as pineapple jam.

Similar to proteins, the addition of chia seeds increased the fiber content of the formulated jams compared to the control. Pineapple jam has a carbohydrate content of approximately 50% of which much is the added sugars with little fiber (Eke‐Ejiofor & Owuno, 2013). Contrastingly, chia seeds contain 42.1% carbohydrates (of which 83% is fiber) and this could be attributed to the increased fiber content of the chia seed‐fortified jams. Chia seeds contain soluble dietary fiber, which is fermented in the colon, and insoluble fibers that form the bulk of the diet. Dietary fiber aids in easy digestion thus aids in bowel movement, providing many health benefits (Anderson et al., 2009). Dietary fiber is thus one of the functional components that could be derived from chia seeds when added to pineapple jam. Chia seeds contain 30.7% fat of which palmitic (7.04%), stearic (2.84%), oleic (7.30%), linoleic (18.89%), α‐ linolenic (63.79%), and arachidic (0.02%) fatty acids are present (Nitrayová et al., 2014). The presence of the essential fatty acids especially α‐linolenic in substantial amounts indicates that consumption of chia seeds can reduce coronary heart diseases and thus its inclusion in pineapple jam confers it with beneficial functional components.

The pH of the control pineapple jam was 3.5 and that of the chia seed‐fortified jams ranged from 3.2 to 3.7, indicating that chia seeds did not affect the pH significantly. The pH of jam is an important parameter which is important in obtaining optimum gel conditions and for preservation of the product (Guerrero & Alzamora, 1998). The sugar in the jams measured as ^°^Brix hardly changed, and the control and the jam formulations had similar figures. This could be due to the fact that much of the carbohydrates (83%) in chia seeds are fibers. The sugar in jam comprises the one in the fruits and the added sugar, which are crucial in the preservation of the product.

Color and texture are crucial sensory attributes on which the consumer acceptance depends. Addition of 6.25% chia seeds did not vary the color and texture of the product significantly, indicating that higher amounts of chia seeds could not be tolerated. For the texture, the low scores where chia seeds were used at higher concentrations could be attributed to the used of whole chia seeds. Use of chia flour could thus give different scores. Flavor of the jam was significantly affected despite chia seed being bland in flavor. This could be attributed to perception as a result of a new ingredient in the jam that consumers are not used to. Spreadability was not affected which could be attributed to the formation of viscous solution of chia seeds. The chia seeds gum (Segura‐Campos, Ciau‐Solís, Rosado‐Rubio, Chel‐Guerrero, & Betancur‐Ancona, 2014) could be contributing to the gelling of jam but not affecting the spreadability of the product.

General acceptability indicated that the control was the most preferred to the chia seed‐fortified jams. There was overall trend that acceptability score was proportional to the amount of chia seeds added. It should be noted that chia seed is regarded as a novel food that has just been introduced in Kenya, with little information on its nutritional information, hence low acceptability scores. However, with sensitization, the product development from chia seeds in the food industry is highly desirable and it could be exploited for nutraceutical food products.

## 5 CONCLUSION

The results obtained indicated that fortification of pineapple jam with chia seeds improves the nutritional value (protein from 0.53% to 8.60% and fiber from 4.83% to 21.02%) without affecting texture. Although chia seeds have been accepted for their functional properties, consumers were not willing to exchange sensory features of pineapple jams for the functional properties and health benefits that are associated with chia seeds. This fact should be considered at the early stages of product development. Nonetheless, the addition of chia seeds at lower amounts (e.g., 6.25% w/w) appeared to have little effects on the sensory characteristics and to be more accepted, implying that in product development, formula optimization in addition to sensitization on the health benefits of functional products is important. Furthermore, the process improvement and shelf life needed to be determined for practical applications.

## CONFLICT OF INTEREST

The authors declare that they do not have any conflict of interest.

## ETHICAL STATEMENTS

This study was approved by the institutional review board of Egerton University. Written informed consent was obtained from all study participants.
